# Vaginoperineal Fistula as a Complication of Perianal Surgery in a Patient with Sjögren's Syndrome: A Case Report

**DOI:** 10.1155/2014/359605

**Published:** 2014-09-10

**Authors:** Kemal Beksac, Mert Turgal, Derman Basaran, Omer Aran, M. Sinan Beksac

**Affiliations:** ^1^Department of General Surgery, Faculty of Medicine, Hacettepe University, Ankara, Turkey; ^2^Department of Obstetrics and Gynecology, Division of Perinatology, Faculty of Medicine, Hacettepe University, Ankara, Turkey; ^3^Department of Obstetrics and Gynecology, Division of Gynecologic Oncology, Faculty of Medicine, Hacettepe University, Ankara, Turkey

## Abstract

Forty-seven-year-old woman with Sjögren's syndrome had been operated on because of transsphincteric perianal fistula secondary to perianal abscess. Vaginal wall injury occurred during the course of the operation and injured tissue was repaired primarily. Three months later, patient suffered from the recurrence of perianal fistula symptoms and fistulectomy was performed once again under antibiotic suppression. Several months later, perineal discharge continued, and, therefore, patient was admitted to the hospital for the third time and a fistulotomy was performed. Two months after the third operation, patient was admitted with leukorrhea and a perineovaginal fistula was detected. This time, not only her surgical problem but also her immune system disorder was considered in the preoperative workup. Then, patient was hospitalized for the fourth time and “fistulectomy/perineoplasty” was performed successfully. We believe that patients with autoimmune disorders with or without medical treatment may have healing problems during the course of surgical processes and therefore such medical problems must be taken into consideration by the surgeons.

## 1. Introduction

Perineovaginal fistula is a rare problem of lower genital tract. It may occur as a complication of episiotomy and perianal diseases [1, 2]. Infections and immune system problems must also be considered as predisposing factors [3, 4]. In this paper, we present a perineovaginal fistula in a patient with Sjögren's disease, as a complication of transsphincteric perianal fistula repair.

## 2. Case Report

Forty-seven-year-old woman with Sjögren's disease had been operated on because of transsphincteric perianal abscess. Vaginal wall injury occurred during the course of the operation and vagina was repaired with primary closure.

Three months later, patient was admitted to hospital with recurrence of the perianal discharge and underwent fistulectomy under antibiotic treatment. Her perineal discharge continued after the operation, and, therefore, patient was hospitalized for the third time and a fistulotomy was performed. Unfortunately, two months later, patient was admitted with leukorrhea and a perineovaginal fistula was detected.

As patient's past medical history was remarkable for Sjögren's disease, a consultation from rheumatology department was requested to stabilize her chronic connective tissue disorder before undergoing definitive surgery. Sjögren's disease and perineal infection were treated with hydroxychloroquine sulfate, methylprednisolone, acetylsalicylic acid, and moxifloxacin. After the initial control of local inflammation, she was discharged with antirheumatic drugs without antibiotics. Two months after the initiation of systemic treatment, she was hospitalized for the fourth time to perform a fistulectomy and perineoplasty.

**Figure 1:**
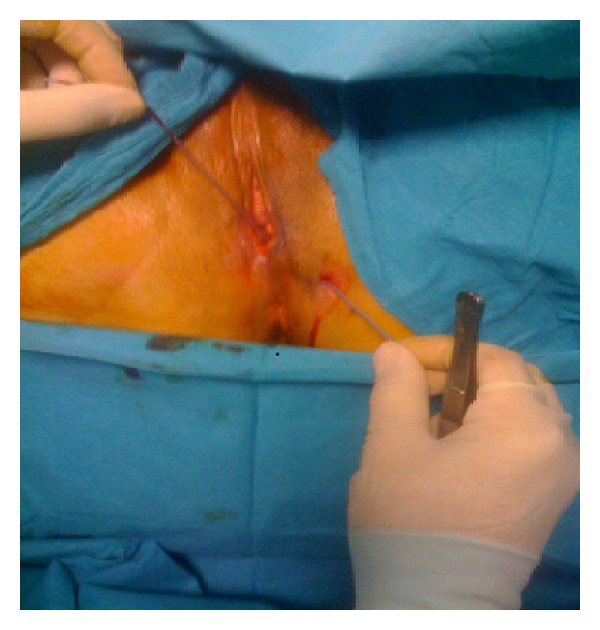
Vaginoperineal fistula.

**Figure 2:**
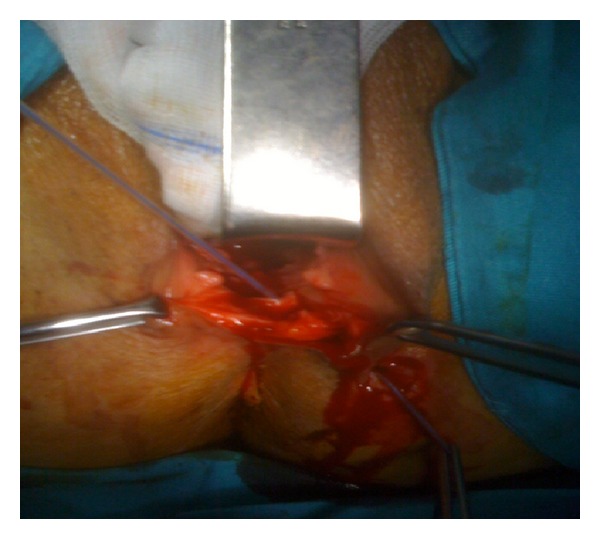
Fistulectomy.

Figure 1 shows the vaginoperineal fistula with a thin catheter passing through it. Vaginal wall and the perineum are opened with appropriate incision and fistula tract is visualized.  Figure 2 shows the fistula just prior to fistulectomy. Entire fistula tract was removed and vaginal mucosa, perineal subcutaneous tissue, and perineal skin were repaired in layers. Recovery was uneventful and patient was discharged on day four. Her systemic antirheumatic treatment continued after the surgery. Follow-up examinations at 6-week, 3-month, and 6-month postop were completely normal.

## 3. Discussion

To the best of our knowledge, this is the first published “vaginoperineal/cutaneous” fistula case as a complication of perianal fistula operation. In the clinical practice, the anatomical and functional integrity of anal sphincter could be disturbed due to episiotomy complication or a tear to the sphincter after vaginal birth [5]. However, in our case, vaginoperineal integrity was disturbed due to perianal fistula operation.

Sjögren's disease may be a predisposing factor for the impaired healing process after surgery. This disease is a rare chronic systemic autoimmune disorder characterized by progressive dryness of the mucous membranes which may be seen with ulcers, and special care may be necessary during the healing processes [6, 7]. Inflammatory bowel disorders such as Crohn's disease could also have a negative impact in the management of perianal fistulas and could interfere with healing processes [3, 4, 8]. In our case, presence of uncontrolled Sjögren's disease could be the most plausible reason of recurrent failures of fistula repair [9]. Therefore, we think that awareness and appropriate treatment of connective tissue disorders could significantly improve the treatment outcomes of surgery for perianal fistulas in which treating the underlying inflammation is mandatory for successful repair [6, 8, 10].

In this case, patient was treated and prepared carefully in terms of her connective tissue problem (Sjögren's disease) together with perioperative antibiotic application. We believe that connective tissue disorders should be taken into consideration during the treatment of recurrent surgical healing problems such as genital and anal fistulas.
